# Multispecies fisheries management of the Sultanate of Oman using bioeconomic model

**DOI:** 10.1016/j.heliyon.2025.e41751

**Published:** 2025-01-08

**Authors:** Sachinandan Dutta, Majid Al Harthy, Saud M. Al Jufaili, Ibtisam Al Abri

**Affiliations:** a Sultan Qaboos University, College of Agricultural and Marine Sciences, Department of Marine Science and Fisheries, Sultanate of Oman; b Sultan Qaboos University, College of Agricultural and Marine Sciences, Department of Natural Resource Economics, Sultanate of Oman

**Keywords:** Maximum economic yield, Maximum sustainable yield, Open access, Effort tax, Entry tax, Sultanate of Oman

## Abstract

A bioeconomic analysis using the Gordon-Schafer surplus production model was conducted on Indian mackerel (*Rastrelliger kanagurta*), Yellowfin tuna (*Thunnus albacares*), Kingfish (*Scomberomorus commerson*), and Indian Oil Sardine (*Sardinella longiceps*) based on data from the Ministry of Agriculture, Fisheries, Wealth, and Water Resources of Oman from 1990 to 2020. The alignment of biological and economic yields with the ideal fishing efforts needed to attain maximum sustainable yield (MSY) and maximum economic yield (MEY) was considered in order to evaluate the economic efficiency of existing fisheries management. The long-term sustainability of Oman's fisheries is improved by this analysis, which identifies inefficiencies in resource use and suggests viable remedies. *Rastrelliger kanagurta* exhibited the highest growth rate (r = 0.260), with similar catchability coefficients (q) between *R. kanagurta* (2.18E-05) and *S. commerson* (2.93E-05), and *T. albacares* (8.48E-06) and *S. longiceps* (8.41E-06). Optimal fishing effort was calculated using the catch per unit effort (CPUE) hypothesis. The effort to achieve maximum sustainable yield (E_MSY_) for *R. kanagurta*, *T. albacares*, *S. commerson*, and *S. longiceps* were estimated at 5982, 6614, 2222, and 6913, respectively. The harvest limit to achieve maximum economic yield (H_MEY_) was 9987.41 tons for *R. kanagurta*, 12928.77 tons for *T. albacares*, 2267.75 tons for *S. commerson*, and 135490.31 tons for *S. longiceps*. A discount rate of 10–20 % was suggested for long-term expansion. The findings aim to guide policymakers in designing sustainable management plans for Oman's fisheries. Stricter fishing rules and the use of industry taxes to control effort levels are two of the study's recommended remedies for overfishing. The objective of these strategies is to maintain long-term sustainability while balancing biological and economic returns, under the supervision of both public and private sector entities.

## Highlights

• A bioeconomic analysis for managing Oman's multispecies fishery.
• The sustainable yield of fishing was used to calculate optimal efforts.
• All species showed consistent catchability coefficients.
• A 10–20 % discount rate was recommended for the long-term expansion of fisheries.
• Taxation and the implementation of overfishing regulations are among the proposed remedies.

## Graphical abstract

**Figure:**
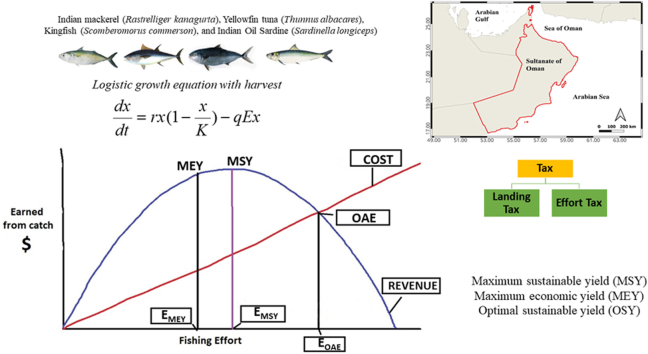

## 1 Introduction

Fisheries play a critical role in the global economy, contributing to food security, employment, and livelihoods [1]. In particular, marine wild fisheries account for approximately 53 % of the total fish food supply, supporting millions of people worldwide, particularly in developing countries [2]. As the demand for fish continues to rise due to population growth and changing dietary preferences, the pressure on marine fish populations has intensified, leading to concerns about the long-term sustainability of fisheries [3]. In response, governments and organizations have emphasized the need for sustainable fisheries management, which balances economic development with the conservation of marine resources [4]. The Sultanate of Oman, situated on the southeastern coast of the Arabian Peninsula, represents a unique case in fisheries management due to its extensive marine resources and economic reliance on fisheries [5]. With a coastline extending over 3165 km and an exclusive economic zone of approximately 536,000 square kilometers, Oman is endowed with abundant marine biodiversity [5].

Despite Oman's vast marine resources, the fisheries sector has faced several challenges, particularly overfishing and inefficient management practices [6]. Over half of Oman's large pelagic species and 68 % of its demersal species are reported to be fully exploited, raising concerns about the sustainability of the country's fisheries [7]. Moreover, Oman's seafood exports yield only half the value per volume compared to the global average, indicating inefficiencies in production and market dynamics [7]. Given these challenges, there is a need for a comprehensive analysis that incorporates both biological and economic factors such as maximum sustainable yield (MSY), maximum economic yield (MEY), and optimal fishing effort to inform sustainable fisheries management [8]. Integrating evaluations of economic efficiency and proposing practical solutions to overfishing, such as industry fees and regulatory enforcement. The goal of these combined insights is to assist policymakers in creating comprehensive and long-term fisheries management plans [9]. The Vision 2040 strategic plan underscores the importance of diversifying Oman's economy, with fisheries identified as a key sector for development [10]. Sustainable fisheries management is crucial not only for economic growth but also for ensuring the long-term viability of marine ecosystems and the livelihoods of coastal communities.

Bioeconomic models offer an effective tool for integrating biological and economic considerations into fisheries management decisions [11,12]. These models simulate the interactions between fish populations, fishing effort, and economic factors, providing insights into optimal management strategies that maximize both ecological sustainability and economic profitability [13,14]. Bioeconomic modeling has been widely used to evaluate the potential outcomes of different management interventions, such as catch limits, effort controls, and seasonal closures [15,16]. By predicting the long-term impacts of various management strategies, bioeconomic models can help policymakers identify sustainable solutions that align with national economic and environmental goals [17] in this uncertain climatic condition. Therefore, it is critical to adjust fisheries management to future climate circumstances because fish species' stable habitats will be essential to guaranteeing long-term economic benefits and sustainable fisheries in the face of climate change [18]. Climate change has an impact on fisheries by affecting ocean temperatures, fish movement patterns, breeding cycles, and habitat availability. Warming waters, for example, might cause changes in species distribution, lower production, and an increased sensitivity to overfishing [18].

Although bioeconomic models have been extensively applied in global fisheries management, there has been limited research in Omani fisheries due to data limitations, a lack of integration, and insufficient regional knowledge. Previous studies have primarily focused on the socioeconomic aspects of fisheries, such as market dynamics, income distribution, and food security [19,20]. However, few studies have addressed the long-term sustainability of fish stocks or developed optimal fisheries management practices based on bioeconomic analysis. Yousuf and Bose [21] estimated reference points for five commercially important demersal species in Oman, but their analysis did not include key pelagic species such as *R. kanagurta*, *T. albacares*, *S. commerson*, and *S. longiceps*, which are among the most economically important species in the country. Furthermore, there is a need for a more integrated approaches such as both biological factors (such as fish stock health) and economic factors (like profitability and resource allocation) that considers both biological and economic factors in the management of Oman's fisheries.

The goal of this study is to fill this gap by applying a bioeconomic model to analyze the sustainability of four key species in Oman's fisheries: *R. kanagurta*, *T. albacares*, *S. commerson*, and *S. longiceps*. These species were selected based on their economic importance and the current concerns about their overexploitation. The study will use catch, effort, and economic data from 1990 to 2020 to estimate the biological, economic, and optimal yields for each species. The bioeconomic model will also be used to evaluate different management scenarios, providing insights into the trade-offs between economic profitability and ecological sustainability. The trader-fisher connection has a substantial impact on fishing methods and jeopardizes the sustainability of the fishery [22]. This emphasizes the necessity of policies that support fishermen's financial autonomy in order to guarantee efficient resource management.

The objectives of this study are: (1) to estimate the biological and economic yields of *R. kanagurta*, *T. albacares*, *S. commerson*, and *S. longiceps*, (2) to determine the optimal fishing effort required to achieve maximum sustainable yield (MSY) and maximum economic yield (MEY) for these species, and (3) to develop management recommendations for ensuring the long-term sustainability of Oman's fisheries. By providing a comprehensive analysis of Oman's marine fisheries, this study aims to support policymakers in developing effective management strategies that align with both economic and conservation goals. This research is timely, given the increasing pressure on global fish stocks and the growing recognition of the need for sustainable fisheries management. As Oman seeks to diversify its economy and reduce its dependence on oil, the sustainable management of its fisheries will be critical to achieving long-term economic and environmental stability. The findings from this study will contribute to the broader understanding of fisheries management in Oman and provide valuable insights for other countries facing similar challenges in balancing economic development with marine conservation.

## 2 Materials and methodology

### 2.1 Data and the study area

**Fig. 1:**
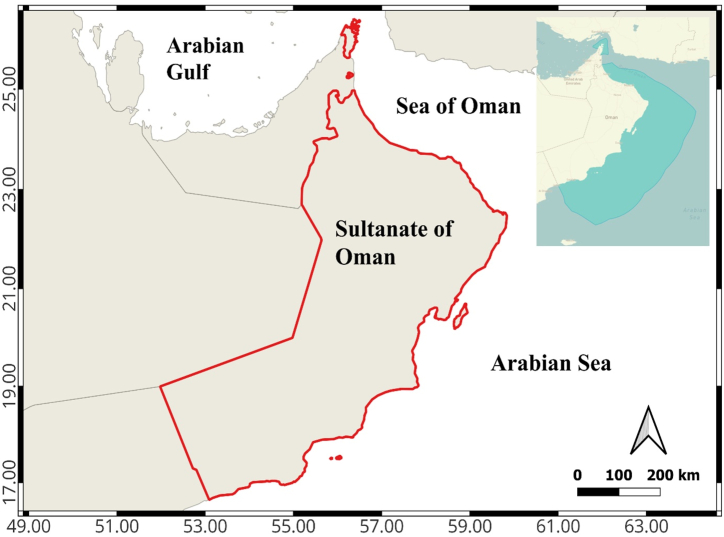
The fishing areas of the Sultanate of Oman.

**Fig. 2:**
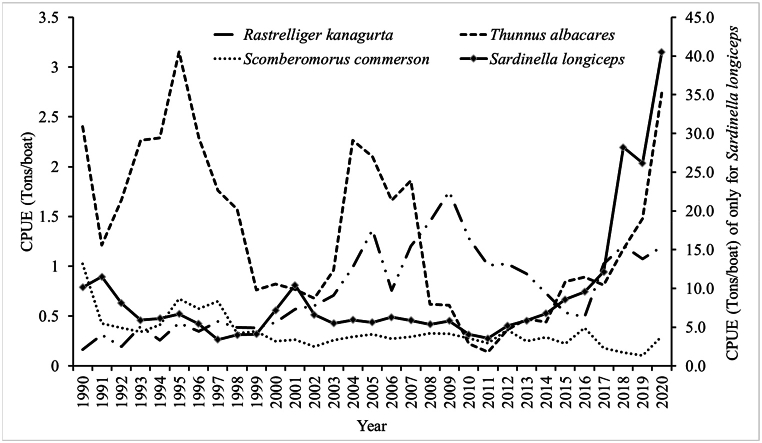
Catch per unit effort (tones/boat) of *Rastrelliger kanagurta, Thunnus albacares, Scomberomorus commerson and Sardinella longiceps* of Sultanate of Oman between 1990 and 2020.

The catch and effort statistics for *R. kanagurta*, *T. albacares*, *S. commerson*, and *S. longiceps* were obtained from the annual ‘Fisheries Statistics Book’ published by the Ministry of Agriculture Fisheries and Water Resources (MAFWR) of Oman between 1990 and 2020 [23], duration of about 31 years along the Omani coast, which includes 3165 km from north Musandam to south Dhofar (Fig. 1). Using time series data of catch and effort, the catch per unit effort (CPUE) data was measured and represented as tons/boat for the four studied fish (Fig. 2). The study was designed to assess data on catch, effort, catch per unit effort, production costs and market prices, maximum sustainable yield in terms of effort, biomass, and harvest. To investigate the open access situation, open access equilibrium for harvest and effort was calculated. Furthermore, the highest economic production and the optimal sustainable yield for different components of *R. kanagurta*, *T. albacares*, *S. commerson*, and *S. longiceps* fisheries were calculated. Market price and fishing cost data for these four species were collected through a survey of fishermen at the Barka and Al Seeb fish markets and fish landing stations between 2021 and 2022. Ice, food, boat crew, transportation, Petrol, and net repairing costs for 280 trips per year were estimated.

Initial proportion (IP), is the ratio of initial to maximum catch in fishery research. The IP was estimated (IP = initial year catch/maximum catch) for *R. kanagurta*, *T. albacares*, *S. commerson*, and *S. longiceps*. The Schaefer model with log-normal error and IP value as an input parameters was used to estimate the following parameters: maximum sustainable yield (MSY), *K* for carrying capacity, *q* for catchability coefficient, *r* for intrinsic growth rate, *R*_*yield*_ for replacement yield, *R*^*2*^ for the coefficient of determination, and final biomass, using the Catch and Effort Data Analysis (CEDA) software version 3.0.1. Furthermore, following the calculation of the parameter in CEDA, the further analysis and parameters (Biological Reference Points (BRPs)) were calculated in the Microsoft Excel program (2016).

### 2.2 Calculating the economic efficiency of the economically important marine fisheries

A deterministic bio-economic model for Oman's economically significant marine fisheries was developed using the Gordon-Schafer surplus production model.

### 2.3 Logistic growth equation (Eq.)

A general biological growth model of a fish stock can be expressed as:

Eq. 1 $$\frac{dx}{dt}=rx\left(1-\frac{x}{K}\right)$$

where x is the biomass, K is the environmental carrying capacity, and r is the intrinsic growth rate. The logistic function grows positively in the interval 0< x < K and has a parabolic growth curve. It is strictly concave from below.

### 2.4 Harvest function

As seen by the following widely used the harvest function H(t) is developed based on the assumption [24] that the catch per unit effort is exactly proportional to the fish density.

Eq. 2 $$\frac{dH}{dt}=qEx$$

where *q* is the catchability coefficient, *E* is the fishing effort. The biomass variable *x* represents here the density of the fish at time *t*.

If the population indicated by Eq. (1) is harvested at a rate *H*(*t*), then Eq. (1) becomes

Eq. 3 $$\frac{dx}{dt}=rx\left(1-\frac{x}{K}\right)-qEx$$

At equilibrium the harvest is

Eq. 4 $$H=qEK\left(1-\frac{qE}{r}\right)=qEK-\frac{q^{2}E^{2}K^{2}}{r}$$

From the catch per unit effort hypothesis we know that,

Eq. 5a $$CPUE=\frac{H}{E}=qK-\frac{q^{2}K}{r}E$$

Eq. 5b CPUE=a+bE

Where, a=qK, $K=\frac{a}{q}$ and $b=-\frac{q^{2}K}{r},b=-\frac{aq}{r},r=-\frac{aq}{b}$

### 2.5 Bio-economic model

The maximum sustainable yield (MSY) of effort, harvest, and biomass have been estimated by differentiating yield with respect to effort and putting the result equals to zero.

Eq. 6a, 6b, 6c $$E_{MSY}=\frac{r}{2q},H_{MSY}=\frac{rk}{4},X_{MSY}=\frac{K}{2}$$

The total cost of fishing effort can be expressed as:

Eq. 7 TC(E)=cE

where *c* is the cost of fishing effort, assuming a constant unit price of harvest, total revenue of the fishery is found by:

Eq. 8 TR(E)=pH(E)

where *p* is the unit price of the economically important marine fisheries.

The economic rent is the difference between total revenue and total cost, so the sustainable economic rent is:

Eq. 9 π(E)=TR(E)−TC(E)

When there is unrestricted fishing or open access, individual fishers typically want to maximize their earnings by putting in the most effort possible. When average revenue of effort (AR(E), or revenue per unit of work, equals average cost of effort (AC(E), this can be accomplished. Thus, when no economic rent is obtained from the fisheries or profit is zero, TR(E) = TC(E) or AR(E) = AC(E) is reached, giving pH(E) = cE, suggesting that

Eq. 10 $$E_{OAE}=\frac{r}{q}\left(1-\frac{c}{pqK}\right)$$

We now replace the effort of open access in Eq. (4) in order to achieve the yield level in open access, which yields

Eq. 11 $$H_{OAE}=\frac{rc}{pq}\left(1-\frac{c}{pqK}\right)\text{.}$$

According to Norman-López and Pascoe [25], the level of landings at which the maximum profit from harvest is obtained is known as the maximum economic yield (MEY). When the cost of an extra unit of effort is equal to the marginal sustainable yield, the long-term economic ideal can be achieved. Assume that the marginal cost per effort is MC(E) and the marginal revenue per effort is MR(E). This means that the point at which MR(E) = MC(E), which yields the greatest difference between total revenue and total costs, is the maximum economic yield (MEY) from the fishery.

Eq. 12 $$\frac{dTR\left(E\right)}{dE}=\frac{dTC\left(E\right)}{dE}\text{.}$$

Thus,

Eq. 13 $$\frac{d}{dE}\left[p\left(qEK-\frac{q^{2}K}{r}E^{2}\right)\right]=\frac{d}{dE}\left(cE\right)$$

It gives:

Eq. 14 $$p\left(qK-\frac{2q^{2}K}{r}E\right)=c$$

Hence,

Eq. 15 $$E_{MEY}=\frac{r}{2q}\left(1-\frac{c}{pqK}\right)\text{.}$$

MEY can be obtained by substituting $E_{MEY}$ in Eq. (4) which gives:

Eq. 16 $$H_{MEY}=\frac{r}{4}\left(K-\frac{c^{2}}{p^{2}q^{2}K}\right)$$

Eq. 17 $$X_{MEY}=\frac{K}{2}+\frac{c}{2pq}$$

From an economic perspective, efficient harvesting is not implied by MSY; rather, efficiency is attained when the rent from the fishery is maximized at the lowest possible cost of harvest, or MEY level. The MEY point is not consistent over time because it is dependent on input costs and seafood prices. When we take time into account as a variable, we can establish dynamic reference points in addition to the static reference points, MSY, MEY, and OAY. The discount rate affects the current cash flow value over time. As a result, the discount rate evaluates the stock quantity that over time maximizes the present value of the resource rent flow. The ideal economic yield biomass is the term used to describe this reference point.

### 2.6 Optimal sustainable yield (OSY)

The equation that maximizes the present value (PV) of the fishery can be expressed as:

Eq. 18 $$\max\;PV=\int_{0}^{\infty }e^{-\delta t}\left(pH\left(t\right)-\frac{c}{qx\left(t\right)}H\left(t\right)\right)dt\text{,}$$

Therefore, the current - value Hamiltonian for this control problem is:

Eq. 19 $$L=pH\left(t\right)-\frac{c}{qx\left(t\right)}H\left(t\right)+\lambda \left[rx\left(t\right)\left(1-\frac{x\left(t\right)}{K}\right)-H\left(t\right)\right]$$

Where λ is the adjoin variable. The Hamiltonian must be maximized for $H_{e}\left[0,H_{\max}\right]$. The study assumes that the control constraints are not binding (the optimal solution does not occur at 0 or $H_{\max}$) and it is called the switching function.

Hamiltonian L is a linear control variable, the optimal control is a mixture of extreme controls and the singular control. To reach the optimal control H(t) that maximizes *L*, it must satisfy the following conditions,

$$H=H_{\max},when,\mu \left(t\right)<0,i.e.\lambda <p-\frac{c}{qx}$$

$$H=0,when,\mu \left(t\right)<0,i.e.\lambda >p-\frac{c}{qx}$$

The optimal stock is:

Eq. 20 $$x^{*}=\frac{K}{4}\left(1-\frac{\delta }{r}\right)+\frac{c}{4pq}+\sqrt{{\left(\frac{K}{4rp}\right)}^{2}{\left(p\left(\delta -r\right)-\frac{cr}{qK}\right)}^{2}+{\left(\frac{K}{4rp}\right)}^{2}\frac{8rp\delta c}{Kq}}\text{.}$$

By using the basic bio-economic input parameters, we also calculate the optimal harvest and optimal effort.

Eq. 21a, 21b $$H^{*}=rx^{*}\left(1-\frac{x^{*}}{K}\right)\;\text{and}\;E^{*}=\frac{H^{*}}{qx^{*}}$$

Thus, the optimal profit is:

Eq. 22 $$\pi \left(p,q,K,r,c,x^{*}\right)=pH^{*}-\frac{c}{qx^{*}}H^{*}\text{.}$$

And the present value of the profit is reduced to:

Eq. 23 $$PV=\int_{0}^{\infty }\left(\pi \right)e\_\delta t=\frac{1}{\delta }\pi$$

### 2.7 Potential solutions to the “open access” problem: tax policies

Let us now assume that the economically important marine fisheries of Oman are in an open access situation and the controlling agencies of these two countries levies a tax (T > 0) in order to achieve *H*_*MSY*_, *H*_*OSY,*_ or *H*_*MEY*_ by incorporating the fishing effort equally *E*_*MSY*_, *E*_*OSY,*_ or *E*_*MEY*_. Here we derived the equations to calculate the different types of tax policies using *H*_*MSY*_ and *E*_*MSY*._ We evaluated the following types of taxes to achieve *MSY*, *OSY,* and *MEY* for the economically important marine fisheries of Oman.

a. Landing tax

If (*T > 0*) the landing tax is to be included for achieving *H*_*MSY*_ or *H*_*MEY*_ by incorporating the fishing effort equally *E*_*MSY*_ or *E*_*MEY*_, then we get the following equation:

Eq. 24a, 24b $$\left(p\;-\;T\right)H_{MSY}=cE_{MSY},\text{which}\;\text{gives}\;T=p-c\frac{E_{MSY}}{H_{MSY}}\text{.}$$

The intercept between *TR* = *(p − T)H* and *TC* = *cE* will give us *E*_*MSY*_*, (p − T)H*_*MSY*_.

b. Effort tax

If (T > 0) the effort tax is to be included to achieve *H*_*MSY*_ or *H*_*MEY*_ by incorporating the fishing effort equally *E*_*MSY*_ or *E*_*MEY*_, then we get the following equation:

Eq. 25a, 25b $$pH_{MSY}=\left(c+T\right)E_{MSY},\text{which}\;\text{gives}\;T=\left[p\frac{H_{MSY}}{E_{MSY}}\right]-c\text{.}$$

The intercept between TR = pH and TC_1_ = (c + T)E will give us E_MSY_, pH_MSY_.

## 3 Results and discussion

The Schaefer model derived key parameters such as intrinsic growth rate (*r*), the Carrying capacity (*K*), the catchability coefficient (*q*), Maximum sustainable yield (*MSY*), the replacement yield (*R yield*), final biomass of *R. kanagurta*, *T. albacares*, *S. commerson* and *S. longiceps*, were presented in Table 1. *Rastrelliger kanagurta* populations of Oman which have the higher growth rate (r=0.260) among all four studied fishes. The catchability coefficient (q) of *R. kanagurta* (2.177E-05) and *S. commerson* (2.93E-05) have almost similar and the similar q was also found in between *T. albacares* (8.48E-06) and *S. longiceps* (8.41E-06). As a result, greater fishing effort may result in a higher total catch [26]. The *R. kanagurta* and *S. longiceps* are frequently found in Omani waters and has the potential to grow in the future due to their high reproductive rates, wide distribution in Omani waters, and the availability of suitable habitats, which support their population increase and sustainable growth in the future. The maximum sustainable yield (MSY) of *R. kanagurta*, *T. albacares*, *S. commerson*, and *S. longiceps* were 10,662.65; 12,991.16; 2574.827 and 135,857.7 tonnes respectively (Table 1).

**Table 1:** Schaefer model estimates of the key parameters for *Rastrelliger kanagurta, Thunnus albacares, Scomberomorus commerson* and *Sardinella longiceps* of Sultanate of Oman.

Model	Species	IP	K (tonnes)	q	r	MSY (tonnes)	R Yield	Final Biomass	r^2^	Error
Schaefer Model	*Rastrelliger kanagurta*	0.05	163745.5	2.177E-05	0.260469	10662.65	7570.42	37782.51	0.4	Log Normal Fit
	*Thunnus albacares*	0.42	463266.9	8.48E-06	0.11217	12991.16	5170.44	51911.62	0.16	
	*Scomberomorus commerson*	1	79007.23	2.93E-05	0.130359	2574.827	214.865	23323.35	0.06	
	*Sardinella longiceps*	0.095	4675602	8.41E-06	0.116227	135857.7	81545.8	859669.6	0.33	

IP=Initial proportion; K = carrying capacity; q = catchability coefficient; r = biological growth rate; MSY = maximum sustainable yield; R yield = recruitment yield.

**Fig. 3:**
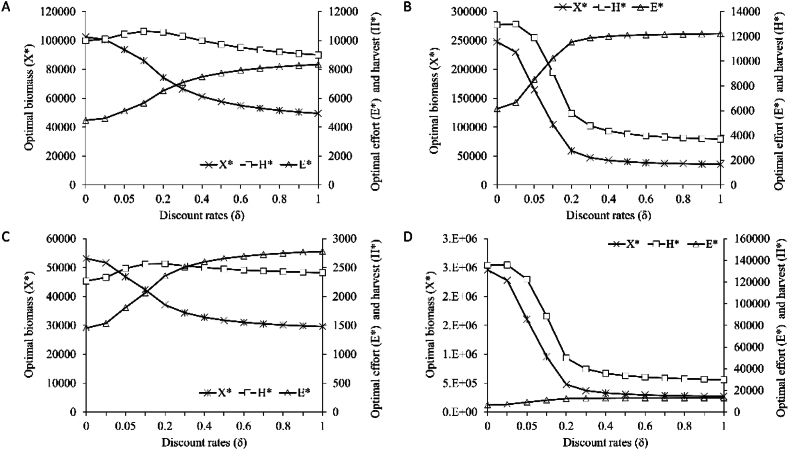
Optimal biomass (X∗) (tonnes), harvest (H∗) (tonnes) and effort (E∗) (no of boats) of (A) *Rastrelliger kanagurta* (B) *Thunnus albacares* (C) *Scomberomorus commerson* and (D) *Sardinella longiceps* of the Sultanate of Oman at different discount (δ) rates.

**Fig. 4:**
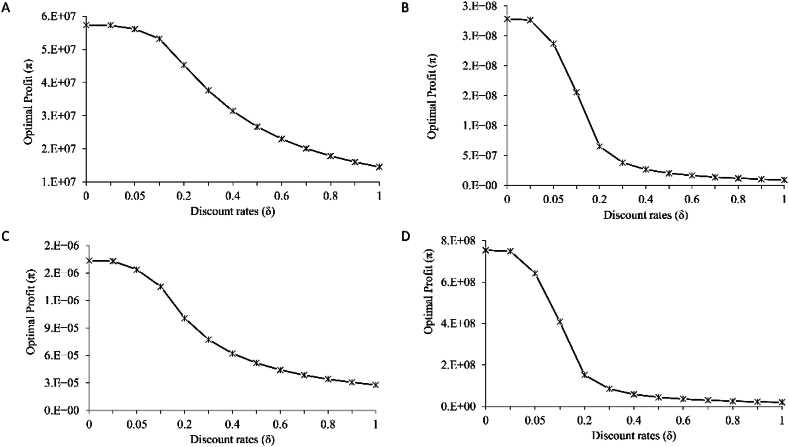
Optimal profit (π) (OMR) of (A) *Rastrelliger kanagurta* (B) *Thunnus albacares* (C) *Scomberomorus commerson* and (D) *Sardinella longiceps* of the Sultanate of Oman at different discount (δ) rates.

The optimal biomass, effort, harvest, and profit of *R. kanagurta*, *T. albacares*, *S. commerson*, and *S. longiceps* fisheries with discount rates ranging from 0 to 100 % was depicted in Fig. 3. When there are no fisheries, biomass and harvest are fairly high, showing that no fishing allows for very high biomass. The ideal biomass was relatively high when the discount rate was zero. When the discount rate was increased the fish biomass continued to fall until it reaches a critical level. The study suggested that fishing boats should be the best fishing effort to obtain maximum economic yield (MEY) for Oman in the scenario of a 10 % discount rate to account for the long-term economic benefits, ensuring sustainable fisheries management while maximizing profitability. Optimal profit (π) of *R. kanagurta*, *T. albacares*, *S. commerson*, and *S. longiceps* in the Sultanate of Oman at different discount rates depicted in Fig. 4. The optimal profit drops as the discount rate rises. It is evident that permitting whole fisheries to be fished leads in a decrease in profit, which gets very low at discount rate 1. A greater discount rate, such as lower fish stock investment, shows a higher return on investment, which may make harvesting more practical in the future [27]. The amount of the discount rate has a substantial influence on the optimal fishing policy [28]. A high discount rate would increase errors of fisheries restrictions (for example, 'Open Entry'), whereas a low discount rate is associated with less intense fishing pressure [24]. The optimal profit continues to fall when the discount rate (δ) is increased from 0 to 100 %; however, a substantial decline in optimal profit was seen from 1 to 20 % discount rate, and then stabilized at >20 percent discount rate. As a result, a discount rate of 10–20 % may be used to accomplish the best effort. According to studies, if δ > 2r, the fishery is deemed depleted, and if δ > r, it is overfished [27,29]. The discount rate of 10–20 % is considered acceptable for this nation. More than a 20 % discount rate may have a detrimental impact on fisheries since it increases catching, whereas, a smaller discount rate reduces fishing pressure.

The effort (E), harvest (H), Biomass (x), total revenue, total cost and profit for the maximum sustainable yield (MSY), the maximum economic yield (MEY), and the optimal sustainable yield (OSY) of all four fish species are depicted in Table 2. The Sultanate of Oman should allow 5981; 6614; 2222 and 6913 numbers of boat for *R. kanagurta*, *T. albacares*, *S. commerson*, and *S. longiceps* respectively to achieve the MSY (Table 2). A dynamic bioeconomic study of an overfished stock can indicate how quickly it must be rebuilt to meet regulatory criteria [30]. The Sultanate of Oman should restrict the catch of *R. kanagurta*, *T. albacares*, *S. commerson*, and *S. longiceps* to 9987.41; 12,928.77; 2267.75 and 135,490.31 tonnes, receptively, to achieve the MEY.

**Table 2:** The effort (E), harvest (H), biomass (x), total revenue, total cost and profit for maximum sustainable yield (MSY), maximum economic yield (MEY), and open access equilibrium (OAE) of all four fish species. (1 OMR = 2.6 US$).

**Species**		**Effort (no of boats)**	**Harvest (tonnes)**	**Biomass (tonnes)**	**Total revenue (OMR)**	**Total cost (OMR)**	**Profit (OMR)**
*Rastrelliger kanagurta*	MSY	5982	10662.65	81872.75	102357373.5	51516277.33	50841096.2
	MEY	4476.32	9987.41	102475.95	95875361.69	38552253.64	57323108.04
	OAE	8952.63	8032.04	10369.55	77104507.29	77104507.29	0
*Thunnus albacares*	MSY	6614	12991.16	231633.45	321452570.5	44553193.84	276899376.7
	MEY	6155.46	12928.77	247685.60	319908806.9	41465666.68	278443140.2
	OAE	12310.92	3351.58	2224.82	82931333.36	82931333.36	0
*Scomberomorus commerson*	MSY	2222	2574.83	39503.62	3814143.989	2634359.441	1179784.549
	MEY	1454.68	2267.75	53145.83	3359268.028	1724607.517	1634660.511
	OAE	2909.37	2328.47	9422.43	3449215.034	3449215.034	0
*Sardinella longiceps*	MSY	6913	135857.80	2337801.00	839138246.4	87286012.84	751852233.6
	MEY	6553.21	135490.31	2459388.43	836868403.5	82746326.99	754122076.5
	OAE	13106.43	26793.52	12647.36	165492654	165492654	0

**Fig. 5:**
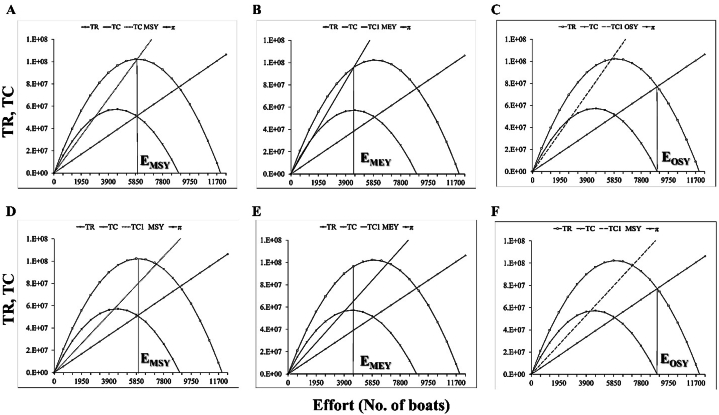
Effort tax required to achieve the (A) maximum sustainable yield (MSY) (B) maximum economic yield (MEY), and (C) optimal sustainable yield (OSY) for *Rastrelliger kanagurta* and landing tax required to achieve the (D) maximum sustainable yield (MSY) (E) maximum economic yield (MEY), and (F) optimal sustainable yield (OSY) for *R. kanagurta* fisheries of the Sultanate of Oman. TR = Total revenue, TC = Total cost, π = Economic rent, TC1 = Total cost after tax.

**Fig. 6:**
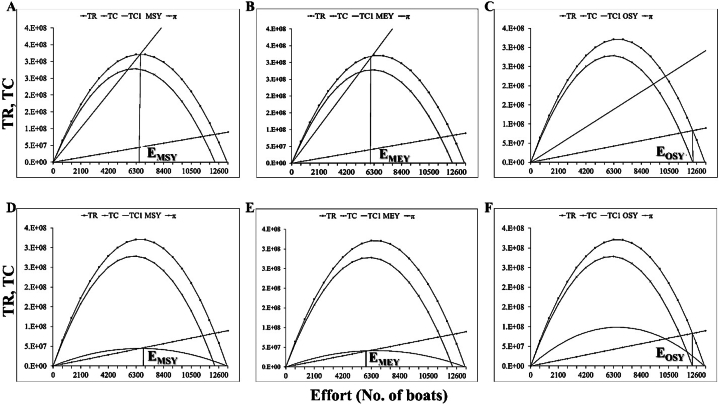
Effort tax required to achieve the (A) maximum sustainable yield (MSY) (B) maximum economic yield (MEY), and (C) optimal sustainable yield (OSY) for *Thunnus albacares* and landing tax required to achieve the (D) maximum sustainable yield (MSY) (E) maximum economic yield (MEY), and (F) optimal sustainable yield (OSY) for *T. albacares* fisheries of the Sultanate of Oman. TR = Total revenue, TC = Total cost, π = Economic rent, TC1 = Total cost after tax.

**Fig. 7:**
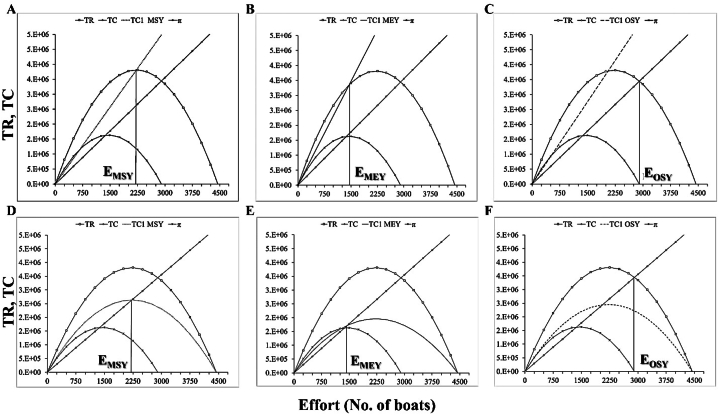
Effort tax required to achieve the (A) maximum sustainable yield (MSY) (B) maximum economic yield (MEY), and (C) optimal sustainable yield (OSY) for *Scomberomorus commerson* and landing tax required to achieve the (D) maximum sustainable yield (MSY) (E) maximum economic yield (MEY), and (F) optimal sustainable yield (OSY) for *S. commerson* fisheries of the Sultanate of Oman. TR = Total revenue, TC = Total cost, π = Economic rent, TC1 = Total cost after tax.

**Fig. 8:**
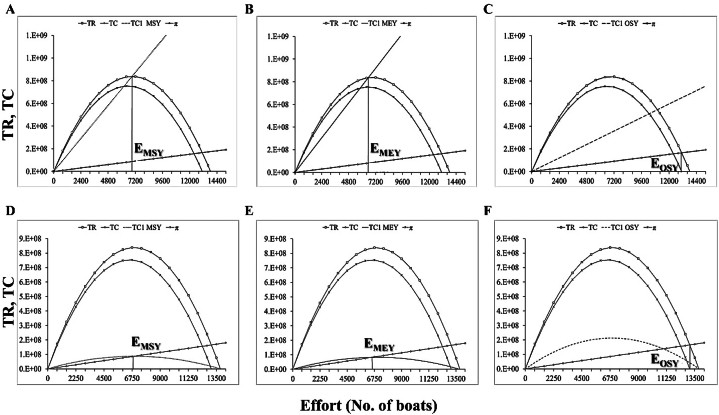
Effort tax required to achieve the (A) maximum sustainable yield (MSY) (B) maximum economic yield (MEY), and (C) optimal sustainable yield (OSY) for *Sardinella longiceps* and landing tax required to achieve the (D) maximum sustainable yield (MSY) (E) maximum economic yield (MEY), and (F) optimal sustainable yield (OSY) for *S. longiceps* fisheries of the Sultanate of Oman. TR = Total revenue, TC = Total cost, π = Economic rent, TC1 = Total cost after tax.

Bioeconomic analysis of the most economically important marine fisheries reveals the optimal economic and ecological fisheries management decisions and potential overfishing remedies [31]. Fig. 5, Fig. 6, Fig. 7, Fig. 8 show the incorporation of effort tax and landing tax for achieving MEY, MSY and OSY of *R. kanagurta*, *T. albacares*, *S. commerson*, and *S. longiceps* in the Sultanate of Oman, respectively. The amount of effort tax needed to achieve the MSY, MEY, and OSY of *R. kanagurta* was depicted in Fig. 5A–C. The Sultanate of Oman's *R. kanagurta* fishery must attain the following: MSY (Fig. 5D), MEY (Fig. 5E), and OSY (Fig. 5F) with a landing tax. The result indicates that the fishing effort for *R. kanagurta*, *T. albacares*, *S. commerson*, and *S. longiceps* fishing are always increasing owing to fisherman's personal well-being, as they seek to maximize their profit, but if they continue to operate in the same pattern, harvest production may decrease. As a result, property rights must be established in order to optimize economic production and to implement optimal management measures in which the marginal net benefit matches the societal marginal cost.

The landing and effort tax for MSY, MEY and OSY of all four studied fish species depicted in Table 3. The issue of open access fisheries might be solved by adopting suitable levies, which would eventually reduce property rights-related difficulties [32]. Thus, by introducing taxes, *R. kanagurta*, *T. albacares*, *S. commerson*, and *S. longiceps* fisheries might be protected from overfishing and sustainably managed [33]. Because taxes would make overfishing less profitable, fishermen would be more inclined to limit their catch to sustainable levels, encouraging ethical fishing methods and safeguarding the fisheries. In general, fisheries management views taxes, licensing, property rights leasing, and seasonal harvesting as effective methods of control [33]. It is advantageous to adopt appropriate tax policies to address property rights issues in open access fisheries. A tax would be preferred for long-term management [34] of these four species since it would prevent the fish species from being exploited. These findings provide guidance for determining the best taxation strategy for a regulatory body that wishes to employ landed fish taxes as a control mechanism to maximize total discounted net revenue from fisheries. A fisheries tax can discourage overexploitation of marine resources and encourage sustainable practices, which would help prevent overfishing [35].

**Table 3:** The landing tax and effort tax for the maximum sustainable yield (MSY), the maximum economic yield (MEY) and the optimal sustainable yield (OSY) of *Rastrelliger kanagurta, Thunnus albacares, Scomberomorus commerson* and *Sardinella longiceps* fisheries of the Sultanate of Oman.

**Species**	**Tax types**	**MSY**	**MEY**	**OSY**
*Rastrelliger kanagurta*	Landing Tax	4768.15	5739.54	5004.75
	Effort Tax	8499.62	12805.87	9380.75
*Thunnus albacares*	Landing Tax	21314.44	21536.70	17147.79
	Effort Tax	41866.93	45235.13	15206.97
*Scomberomorus commerson*	Landing Tax	458.20	720.83	527.20
	Effort Tax	530.94	1123.72	655.09
*Sardinella longiceps*	Landing Tax	5534.11	5565.87	4609.33
	Effort Tax	108763.26	115076.67	37135.75

*Rastrelliger kanagurta*, *T. albacares*, *S. commerson*, and *S. longiceps* are the most highly valued fish in the Sultanate of Oman and they one of the most commercially important species in the Gulf Cooperation Council (GCC) area. Taxes (both landing and effort) should be applied for the studied fishery to further grow this industry on a sustainable basis. Controlling fishing effort and catch is the most prevalent management approach employed in the fisheries sector. Fishing and harvesting are often governed by gear restrictions, closed seasons, restricted entrance, or quota allocation [36]. The fishery industry should adopt numerous regulations, including those for resource conservation, exploitation, and management, in order to better utilize Oman's fisheries for *R. kanagurta*, *T. albacares*, *S. commerson*, and *S. longiceps*. Moreover, a number of strategies are needed, including a prohibition on harvesting during the peak spawning season, mesh size restrictions, providing alternative resources or livelihoods to the fishers during the worst period, and a conservation movement. Catch and effort should be restricted to the level of profit maximization, i.e up to maximum economic yield. Moreover, a discount rate of 10–20 % seemed preferable for the long-term growth of fishery. This study could assist policymakers in developing effective management plans to ensure the sustainability of marine fisheries in the Sultanate of Oman. The fisheries might be further exploited on a sustainable basis by imposing taxes on the industry and adopting different fishing laws, which should be enforced and supervised by various government and private sector committees. In order to discuss and lay out plans for trans-boundary management of fishery resources based on modern practices in fisheries science and management, expert groups from international organizations, intergovernmental autonomous bodies, non-governmental organizations, the private sector and development partners, fisheries associations, academia, civil society, and local and government representatives could all be involved in the development of multi-stakeholder strategies [37,38]. It is also important to address the issues of climate change disturbances and pollution to ensure the long-term viability of trans-boundary fisheries management strategies. To be sufficient for reaching resource management objectives, the management of *R. kanagurta*, *T. albacares*, *S. commerson*, and *S. longiceps* should apply the same rules and regulations throughout the coastal states. In future the study related to tagging to assess stock structure, estimate natural and fishing mortality, on movement patterns, and impose yearly taxes will improve the fish stock.

In conclusion, using the Gordon-Schaefer surplus production model, a bioeconomic analysis of Oman's major fish species revealed the need for more sustainable fisheries management. In order to maximize sustainable and profitable harvests, the study placed a strong emphasis on fishing effort optimization. Industry taxes, which raise operating expenses for fishermen who go above reasonable limits, are suggested as a way to curb excessive fishing activity. Stricter fishing laws are also suggested in order to guarantee adherence and lessen overexploitation. These laws include gear limitations, seasonal closures, and monitoring systems. Effective fisheries management requires a trans-boundary management initiative in addition to these laws, guaranteeing sustainable practices across shared marine resources and ecosystems [39]. For these policies to be more effective, government agencies must coordinate their oversight and enforcement, and they must work with private parties. These findings give policymakers a starting point for creating plans that strike a balance between Oman's marine resources preservation and economic growth.

## CRediT authorship contribution statement

**Sachinandan Dutta:** Writing – original draft, Software, Methodology, Funding acquisition, Formal analysis, Data curation, Conceptualization. **Majid Al Harthy:** Writing – review & editing, Software, Formal analysis. **Saud M. Al Jufaili:** Writing – review & editing, Visualization. **Ibtisam Al Abri:** Writing – review & editing, Methodology, Conceptualization, Funding acquisition.

## Ethical approval

Not applicable.

## Consent for publication

All of Author consent for publication.

## Availability of supporting data

Data will be available upon request.

## Funding

This work is based upon research funded by 10.13039/501100004351Sultan Qaboos University under Internal Grant project No. IG/AGR/FISH/21/01. This research work was also supported by HMTF Strategic Research Grant [SR/AGR/ECON/23/01] at Sultan Qaboos University, entitled “Vitalizing and Boosting Fisheries Sector's Contribution Toward Oman's Sustainable Economic Development and Diversification: From Wealth Consuming to Wealth Generating Sector”.

## Declaration of competing interest

The authors declare that they have no known competing financial interests or personal relationships that could have appeared to influence the work reported in this paper.
